# Sodium-Glucose Linked Transporter Inhibitors as a Cause of Euglycemic Diabetic Ketoacidosis on a Background of Starvation

**DOI:** 10.7759/cureus.10078

**Published:** 2020-08-27

**Authors:** Abubakar Tauseef, Muhammad Sohaib Asghar, Maryam Zafar, Noman Lateef, Joseph Thirumalareddy

**Affiliations:** 1 Internal Medicine, Creighton University, Omaha, USA; 2 Internal Medicine, Dow International Medical College, Dow University Hospital, Dow University of Health Sciences, Karachi, PAK; 3 Medicine, Dow International Medical College, Dow University Hospital, Dow University of Health Sciences, Karachi, PAK; 4 Hospital Medicine, Creighton University, Omaha, USA

**Keywords:** diabetes mellitus, euglycemic, sglt-2, diabetic ketoacidosis, glucose, insulin

## Abstract

We present a case of a male diabetic patient with one of the most well-known major complications of diabetes mellitus (DM), i.e., diabetic ketoacidosis (DKA). The finding of euglycemic DKA, or DKA with blood glucose levels of less than 200 mg/dL, is a rare occurrence especially in patients with type II DM. He presented with hypotension and increased anion gap metabolic acidosis on a background of keto diet for weight loss and recent use of sodium-glucose linked transporter inhibitors. He was successfully managed with dextrose water, insulin infusion, and potassium replacement. A ketogenic diet consists of high fat, low carbohydrate, and adequate protein regimen that sends the body into a state of starvation in which high glucagon and low insulin levels lead to the activation of other counter-regulatory hormones, such as epinephrine and cortisol, that causes a rise in the level of free fatty acids in the blood increasing ketone body production. Rarely, sodium-glucose linked transport inhibitors can also cause DKA, with euglycemia instead of hyperglycemia. The finding of plasma glucose levels within normal range prompted patients to maintain and sometimes even lower their insulin dose; even their providers were often misled by the euglycemia that resulted in delayed diagnosis and treatment. Thus, it is imperative to stay aware of the possible clinical presentations in order to intervene in a timely manner and prevent further progression and complications.

## Introduction

One of the most well-known major complications of diabetes mellitus (DM) is the development of diabetic ketoacidosis (DKA). DKA most commonly presents in individuals with type I DM with raised plasma glucose levels in the characteristic triad of hyperglycemia, ketosis, and metabolic acidosis [1]. The finding of euglycemic DKA, or DKA with blood glucose levels of less than 200 mg/dL, is a rare occurrence especially in patients with type II DM [2-4]. In our case, the patient was a type II diabetic who followed a ketogenic diet for three weeks and lost 17 lbs. A diagnosis of type I DM was ruled out, and the patient was found to be in euglycemic ketoacidosis likely triggered by his dietary habits in combination with the use of empagliflozin.

A ketogenic diet consists of high fat, low carbohydrate, and adequate protein regimen that sends the body into a state of starvation in which high glucagon and low insulin levels lead to the activation of other counter-regulatory hormones, such as epinephrine and cortisol, that causes a rise in the level of free fatty acids in the blood increasing ketone body production [5,6]. The risk for the development of metabolic ketoacidosis increases as glycogen is depleted to maintain normal plasma glucose levels and ketones become the predominant means of metabolism in the body [7].

Sodium-glucose linked transport inhibitors work by preventing the absorption of glucose through the nephrons, hence enhancing the excretion of excess glucose through the urine [8]. It has been approved for usage in treating DM since 2013. Recently, it has also been approved to treat heart failure in type II DM patients with a reduction in cardiovascular complications and death [9].

## Case presentation

A 61-year-old male with a significant past medical history of type II DM, being managed by metformin and insulin for the last 10 years, prescribed empagliflozin two months back, presented to the emergency department with hypotension. He mentioned that he started a keto diet three weeks back and lost 17 lbs. He denied polyuria, polydipsia, polyphagia, headache, nausea, vomiting, headache, altered level, and loss of consciousness. His physical examination was unremarkable. He was started on fluid replacement with Ringer lactate, and workup for blood sugars, complete blood count (CBC) with differential, basal metabolic profile (BMP), lactic acid, arterial blood gases, beta-hydroxybutyrate, serum osmolarity, and procalcitonin were sent.

Laboratory investigation showed increased anion gap metabolic acidosis with a pH of 7.11 (normal: 7.35-7.45), serum bicarbonate (HCO_3_) of 7 mEq/L (normal: 20-28) and an anion gap of 20 mEq/L (normal: 8-16), his lactate level came out to be 6.1 mg/dL (4.5-19.8), blood sugars were 90 mg/dL (normal: 80-120), serum osmolarity of 310 mOsm/kg H_2_O (normal: 285-295), and beta-hydroxybutyrate of >4.5 mmol/L (normal: 0.4-0.5). The rest of the laboratory investigations were unremarkable. He got diagnosed with DKA but with completely normal serum glucose levels, it could be secondary to his keto diet/starvation or due to the use of empagliflozin. He got transferred to the Intensive Care Unit (ICU) for further management and started on 5% dextrose water (D5W)/0.45% normal saline with 20 mEq of potassium fluids with insulin infusion at a rate of 1 unit/kg/hr, and metformin and empagliflozin were discontinued. His blood sugars were monitored every two hourly which mostly stayed between 90 and 100 mg/dL. Therefore, his urine was sent for ketones which returned positive. The chest X-ray was unremarkable. The endocrinology department was consulted, and they recommended continuing the same management plan with workup for ruling out type I DM, which was negative. His management was continued for 24 hours with a very little improvement, as his pH increased to 7.20 with an anion gap of 15 mEq/L, HCO_3_ of 11 mEq/L, lactic acid of 4.7 mg/dL, blood sugars of 95 mg/dL, and serum osmolarity of 305 mOsm/kg H_2_O. He was continued on the same management plan for another 24 hours but with an increased rate of fluid to 200 mL/hr. Anion gap was closed at 8 mEq/L, with a pH of 7.37, HCO_3_ of 20 mEq/L, lactate came down to 0.7 mg/dL, blood sugars stayed between 90 and 100 mg/dL, and serum osmolarity decreased to 295 mOsm/kg H_2_O.

The patient was started on subcutaneous insulin detemir with an overlap of an insulin infusion for two hours and infusion was weaned off afterward. A dietician was consulted, who discontinued his keto diet and started him on a regular diet. The patient was discharged home with subcutaneous insulin detemir daily and metformin 1,000 mg BID with a follow-up in one week with the critical care team and endocrinologist. His follow-up was unremarkable with blood sugar levels of 110 mg/dL in the last one week and no acidosis. The patient was counseled regarding all the options for reducing his weight which was his concern and was educated about how to implement keto diet in his life in order to prevent any further episodes of severe acidosis.

## Discussion

Sodium-glucose linked transport inhibitors, such as empagliflozin, are approved for clinical use in the treatment of type II DM in adults; they induce an increase in the excretion of glucose by the kidneys resulting in lower blood glucose levels [8]. Some adverse effects associated with these inhibitors include dehydration, increased cholesterol levels, yeast infections, and kidney problems. Rarely, they can also cause DKA, and interestingly, many of those cases revealed euglycemia instead of hyperglycemia [9].

A few studies report euglycemic DKA due to a low-carbohydrate diet, including one conducted in Japan in which the patient was on canagliflozin and after severely restricting her carbohydrate intake for six days, presented with progressive dyspnea and altered mental status; laboratory investigations revealed severe ketoacidosis and euglycemia [10]. Another case in Taiwan involved a type II diabetic patient on dapagliflozin with a history of poor oral intake for one week attributed to severe toothache who came with the complaints of weakness, dyspnea, nausea, vomiting, and mild abdominal pain; severe metabolic acidosis with an elevated anion gap was discovered upon further investigation [11].

Other causes of euglycemic DKA include pregnancy, cocaine abuse, pancreatitis, cirrhosis, and sepsis; thus, normal plasma glucose levels should not be sufficient to rule out the possibility of DKA in a patient especially if he/she is on sodium-glucose linked transport inhibitors [12,13]. Although the risk of euglycemic DKA in type II DM associated with sodium-glucose linked transport inhibitors is low enough to have an “acceptable frequency” according to the American Diabetes Association, it may be higher in patients with long-standing type II DM during periods of stress such as post-surgery and prolonged starvation [14]. The first case series of euglycemic DKA in the United States found that the common feature in the nine cases presented was that despite the development of ketoacidosis, the finding of plasma glucose levels within normal range prompted patients to maintain and sometimes even lower their insulin dose; even their providers were often misled by the euglycemia that resulted in delayed diagnosis and treatment [15]. Thus, it is imperative to stay aware of the possible clinical presentations in order to intervene in a timely manner and prevent further progression and complications.

## Conclusions

Our case presented with hypotension and increased anion gap metabolic acidosis on a background of controlled type II diabetes with the use of sodium-glucose linked transport inhibitors and keto diet. With blood sugar levels within normal limits, he was managed as a case of euglycemic ketoacidosis, which is a rare side effect of sodium-glucose linked transport inhibitors as well as reported in paucity with starvation in known diabetics. He was successfully managed with D5W/0.45% normal saline, insulin infusion, and potassium replacement.
